# Redevelopment of the Predict: Breast Cancer website and recommendations for developing interfaces to support decision‐making

**DOI:** 10.1002/cam4.4072

**Published:** 2021-06-21

**Authors:** George D. Farmer, Mike Pearson, William J. Skylark, Alexandra L. J. Freeman, David J. Spiegelhalter

**Affiliations:** ^1^ Winton Centre for Risk and Evidence Communication University of Cambridge Cambridge UK; ^2^ Division of Neuroscience and Experimental Psychology University of Manchester Manchester UK; ^3^ Department of Psychology University of Cambridge Cambridge UK

**Keywords:** breast cancer, cancer management, prognosis, risk assessment, translational research

## Abstract

**Objectives:**

To develop a new interface for the widely used prognostic breast cancer tool: Predict: Breast Cancer. To facilitate decision‐making around post‐surgery breast cancer treatments. To derive recommendations for communicating the outputs of prognostic models to patients and their clinicians.

**Method:**

We employed a user‐centred design process comprised of background research and iterative testing of prototypes with clinicians and patients. Methods included surveys, focus groups and usability testing.

**Results:**

The updated interface now caters to the needs of a wider audience through the addition of new visualisations, instantaneous updating of results, enhanced explanatory information and the addition of new predictors and outputs. A programme of future research was identified and is now underway, including the provision of quantitative data on the adverse effects of adjuvant breast cancer treatments.

Based on our user‐centred design process, we identify six recommendations for communicating the outputs of prognostic models including the need to contextualise statistics, identify and address gaps in knowledge, and the critical importance of engaging with prospective users when designing communications.

**Conclusions:**

For prognostic algorithms to fulfil their potential to assist with decision‐making they need carefully designed interfaces. User‐centred design puts patients and clinicians needs at the forefront, allowing them to derive the maximum benefit from prognostic models.

## Graphical abstract

For prognostic algorithms to fulfil their potential to assist with shared decision‐making they need carefully designed interfaces. User‐centred design puts patients and clinicians needs at the forefront, allowing them to derive the maximum benefit from prognostic models.

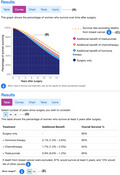

## 1 INTRODUCTION

Around 55,000 women are diagnosed with invasive breast cancer each year in the United Kingdom,^1^ with an estimated 2 million new cases each year worldwide.^2^ These women, along with their healthcare professionals, need to make potentially life‐altering decisions about their treatment. In order to inform those decisions, they need comprehensible and balanced information about the potential risks and benefits of the different treatment options.^3^

In 2010,^4^ a prognostic model called PREDICT was developed to estimate the survival benefits of different adjuvant (post‐surgery) therapies for breast cancer. Individualised estimates are made on the basis of inputs describing the patient and their cancer. The PREDICT algorithm was embedded into a publicly available website, Predict: Breast Cancer, designed primarily for clinicians.

Whilst the PREDICT statistical model has been extensively validated,^5^, ^6^, ^7^, ^8^, ^9^, ^10^ the initial interface that allowed public access had not been designed or tested for comprehension and usability. It is well recognised that the ways in which numbers and evidence are presented can have a large impact on the audience's perception of risks and benefits, and on decisions made as a result.^11^ Careful design of the outputs of risk prediction algorithms is, therefore, necessary. Good design should improve comprehension, shared decision‐making and standardisation of treatment. We, therefore, set out to redesign the Predict: Breast Cancer interface, using the principles of user‐centred design (UCD),^12^ combined with knowledge from the literature on the visual communication of risk.^11^, ^13^ This process resulted in a new interface collaboratively developed with patients and clinicians.

The UCD process is widely used in industry and considered best practice when designing interactive systems. The use of UCD in a medical context is less common but increasingly recognised as important.^14^, ^15^, ^16^ We outline how we applied the UCD process to the Predict: Breast Cancer interface, and develop recommendations that may help others ensure prognostic models maximise their potential to help with decision‐making.

Post‐redesign, the site is delivering over 30,000 sessions per month. It is recommended by the National Institute for Health and Care Excellence (NICE) in the United Kingdom and endorsed by the American Joint Committee on Cancer.^17^

## 2 METHODS

This study was approved by the University of Cambridge Psychology Research Ethics Committee (PRE.2016.103). Participants gave informed consent before taking part.

**FIGURE 1:**
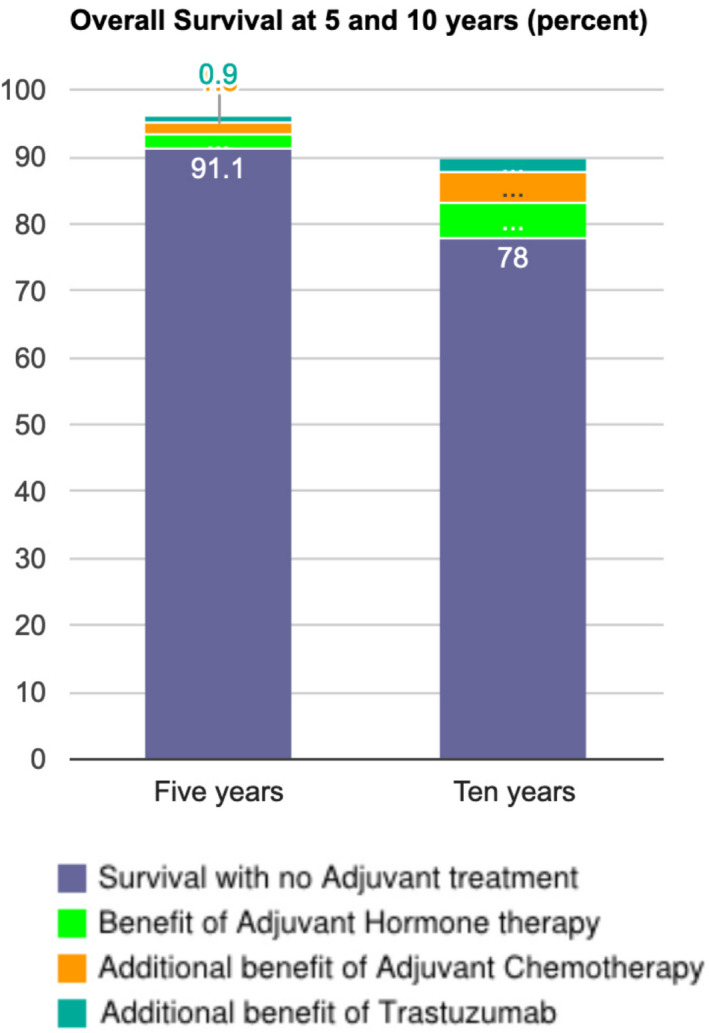
The original interface as it appeared on the Predict: Breast Cancer website. Hovering over a segment would reveal a pop‐up display showing the increase in survival. The increases were also displayed as text below the chart

The original interface allowed of parameters describing the patient and their cancer. The user could then select a combination of treatments that are available after surgery to further treat the cancer. These included hormone therapy, chemotherapy and trastuzumab (a targeted antibody treatment). Other treatments are available but were not included in Predict: Breast Cancer at the time. After entering parameters and selecting treatments, the user was presented with predicted survival at 5 and 10 years in the form of text and a stacked bar chart. The display included a breakdown of how each treatment contributed to the estimated survival rate (see Figure 1).

### 2.1 Background research

To understand more about patient use of Predict: Breast Cancer, the term 'Predict' was searched for on the fora of prominent breast cancer charities. There were not sufficient data for a detailed analysis although the comments found provided a valuable insight into patients' unmediated experience of the Predict: Breast Cancer website.

We conducted a focus group with members of the general public. Women from the Cambridge area (*n* = 7, Mean age = 46, *SD* = 13) were recruited via online community message boards. Participants' education level spanned GCSE level (1), A level (2), degree level (3) and post‐graduate (1). Household income categories spanned less than £10,000 per annum to £51,000 to £60,000 per annum. We sampled the general population to obtain the perspective of women encountering prognostic breast cancer information for the first time. Participants attended a session lasting 90 min that followed a semi‐structured agenda. Participants discussed their expectations of a prognostic website, how comprehensible different visualisations were, and features not currently available that they would find desirable. An audio recording of the focus group was made, and notes taken by the two researchers were present. Notes and recordings were subsequently analysed for comments and suggestions that enabled us to produce high‐level requirements for the design of a new interface.

To validate and expand our feedback, we also carried out an online survey (*n* = 50, mean age = 37, *SD* = 10) with members of the public recruited from a national participant pool (prolific.co). Participants' household income spanned below £10,000 per annum to over £100,000 per annum. The modal income category (*n* = 12) was £20,000–£30,000. Participants' education level spanned none (1) to doctoral (2). The modal education level was secondary/high school (22). Participants were given input parameters, asked to enter these into Predict: Breast Cancer and then comment on the appearance, perceived trustworthiness of the site and on how the results were displayed. The survey respondents also rated Predict: Breast Cancer on appearance, trustworthiness and interpretability of the outputs on a 5‐point scale ranging from 'completely disagree' to 'completely agree'. Free‐text responses to the online survey were aggregated and given an initial code to identify comments relating to a common theme. These were further summarised to produce broad design requirements.

To understand more about clinician use of the tool we observed a multi‐disciplinary team meeting (MDT) at the Cambridge Breast Unit. MDTs were identified during the background research as a critical use case for Predict: Breast Cancer. During MDTs a team of clinicians collectively decide on which adjuvant treatments to recommend to a patient, and Predict: Breast Cancer is often used to determine whether treatments offer enough benefit to merit being recommended. Notes from the observation of the MDT were reviewed to derive high‐level design requirements specific to that setting.

We also held a focus group with 18 clinicians including 11 breast oncologists (specialisms included radiotherapy and chemotherapy) at the Cambridge Breast Unit, addressing how they used the site, issues that they had with the current version and their opinions on how it could be improved. The clinician focus group was not recorded but was attended by four researchers whose meeting notes were collated and compared to identify issues within the remit of the interface redesign.

To validate our clinician feedback, we also surveyed attendees at the UK Breast Cancer Group (UKBCG) meeting in November 2016. Respondents (*n* = 75) were from 44 institutions across England, Scotland, Wales and Northern Ireland. All respondents indicated that they had used Predict: Breast Cancer before. The majority of respondents were consultants (47) but also included surgeons, research fellows and registrars. The survey asked respondents about the contexts they used Predict: Breast Cancer in, and the devices they used to access it. Respondents could indicate features from other prognostic tools that they would like included and were also asked whether they agreed with a list of potential new predictors and outputs. Free‐text fields were included to allow respondents to expand on any of their answers.

In all cases we adopted a qualitative descriptive approach^18^ which minimises interpretation by the researchers, instead focussing on recording and summarising participants' contributions. This is an efficient approach suitable for background research in UCD, where the objective is to generate a broad range of issues that should be considered when designing a new interface.

### 2.2 Iterative development

We developed prototypes informed by the background research. Wireframes were used to encourage feedback that included substantial changes to the design. A wireframe is a sketched interface used to communicate potential functions and layouts. The sketched appearance avoids participants assuming the design has already been finalised and instead encourages them to suggest different layouts and features. Later iterations with increased functionality and realism resulted in incremental feedback until a final prototype was ready for launch.

Using wireframes we obtained feedback and suggestions for improvement from breast cancer clinicians at Addenbrooke's hospital (6 oncologists). To broaden the feedback, we also visited clinicians at Hillingdon hospital (3 cancer nurse specialists and 2 oncologists). For both sites participants were shown the wireframes and given a demonstration of example interactions that it would allow (e.g., selecting different timeframes over which to see the predictions of the algorithm). Participants were then invited to discuss problems, benefits and alternative solutions. These meetings were attended by at least 2 researchers and notes were collated and compared to produce design requirements for the next iteration––a functioning prototype.

Based on the wireframe feedback, a functioning prototype was made available to participants for usability evaluation. These included 7 breast oncologists who had indicated that they were willing to be contacted when we surveyed the UK Breast Cancer Group (UKBCG) 2016 conference, and 12 breast cancer patients (mean age = 53, *SD* = 10) were recruited from a pool of participants who had previously worked with our centre. These patients had previously been diagnosed with breast cancer, but were beyond the stage where Predict: Breast Cancer is typically used. Patients' household income ranged from the £21,000 to £30,000 category to the over £91,000 category. Education levels spanned GCSE (1), A‐level (2), degree (6) and post‐graduate (3). Participants were asked to interact with the website whilst software recorded video, audio and mouse movements. Each participant undertook a pre‐set task of entering data and interpreting the results, as well as some unstructured interaction in which they were asked to use the site as they would normally.

Notes and reviews of recordings were used to produce a table for each participant detailing issues encountered and their location within the website. User interface updates to address consistent issues were made on an ongoing basis such that later participants saw changes made as a result of earlier feedback.

In the final stage we employed a graphic designer to develop a professional standard design for the website based on the participant feedback. This prototype was made available to the patient advocacy group Independent Cancer Patients' Voice, who reviewed the site before the final iteration and launch.

## 3 RESULTS

### 3.1 Background research

Table 1 summarises the principal issues and design requirements identified from the background research with non‐clinicians. These were derived by grouping forum comments and focus group notes into broad themes. The comments shown in Box 1 were chosen to be representative of recurring sentiments found on the breast cancer fora.

**TABLE 1:** Major design requirements derived from background research with patients and the public

Source	Issue	Requirements
Patient fora	Fear of poor prognosis	Address language, appearance and provide links to support
	Other tools provide different predictions	Guidance on interpreting prognostic statistics
	Averages not perceived as relevant to individuals	Guidance on interpreting prognostic statistics
	Predictions must be based on old data to provide long term predictions	Guidance on interpreting prognostic statistics. FAQ section.
	Treatment benefit sometimes perceived to be surprisingly small	Ability to trade‐off benefits and adverse effects
Public survey *n* = 50, mean age = 37, *SD* = 10	Appearance too basic and impersonal	Address language, appearance and provide links to support
	NHS critical to instilling trust	More prominence to NHS branding
	Want more information about side effects	Ability to trade‐off benefits and adverse effects
	Outputs difficult to read/interpret	Improved visualisation of results
	Averages not perceived as relevant to individuals	Guidance on interpreting prognostic stats
Public focus group *n* = 7, mean age = 46, *SD* = 13	Fear of poor prognosis	Address language, appearance and link to support
	Desire to take part in decision‐making	Facilitate communication between patients and clinicians
	Technical information important but incomprehensible	Rewrite technical information to improve comprehension
	Preference for abstract visualisations. Icons representing people too upsetting	Consider emotional impact in design of graphics and labelling
	Want information on side effects	Ability to trade‐off benefits and harms

BOX 1 **Illustrative comments about the Predict: Breast Cancer tool on patient forums. Note comments are paraphrased to preserve the original posters' anonymity.**

1. Why would anyone want to know predicted survival rate? Living through this is bad enough.

2. All these tools are based on outdated figures anyway. Someone diagnosed today is likely to do better than someone diagnosed 10 or 15 years ago.

3. I like the CancerMath tool because it gives much more optimistic chances (Common comment, but N.B. this is because it does not include non‐breast cancer mortality).

4. It will come back or it won't, percentages don't make sense.

5. The benefit of the treatment was so small, very surprised.

The public survey data resulted in 249 free‐text comments. These were given an initial code, for example, the comments: *'provides statistical info but fees very clinical,* *[…]at a time when you are dealing with cancer you might appreciate a more personal feel'*. and '*It may provide help and reassurance to see actual numbers. However I do think it's a little impersonal ‐ especially to a potentially vulnerable cancer sufferer*' were initially coded as 'impersonal'. The impersonal code was further summarised along with similar codes to make up the 'Appearance too basic and impersonal' issue in Table 1. Participants' Likert ratings were largely positive about the site with the majority selecting a better than neutral rating for each of the questions (see Table S1 ).

After participants had entered the parameters we gave them, we asked them to report the predicted survival for women who: (1) have no adjuvant treatment, (2) have only hormone therapy and (3) have hormone therapy and chemotherapy. Ninety‐two per cent answered question 1 correctly, however only 58% and 46% got questions 2 and 3 correct, respectively. Errors were due to participants reporting the increase in survival associated with a treatment rather than the total survival. These data point to a possible misinterpretation of the stacked bar charts such that each segment is viewed as an alternative treatment rather than being in addition to the segments below it (see Figure 1 for an example of the display). We should acknowledge, however, that these errors could equally be a misinterpretation of the question rather than the chart, although subsequent research does point to problems in interpreting the chart.^19^

From the three sources of non‐clinician feedback, we derived the following overarching themes.

***Fear of a poor prognosis***: Many participants indicated that they would be put off using a prognostic tool for fear of receiving worse news than they expected. For some, there was a sufficiently strong sense of foreboding that using the tool would simply be too frightening (e.g., Box 1 comment 1).

***Appearance and trustworthiness***: Participants in the online survey and focus group identified that the site appeared 'clinical', 'cold' and 'basic'. The clinical tone resulted in a perceived lack of empathy for the user whilst the basic style risked a lack of authority. Focus group participants also indicated that whilst icon arrays were a good communication device in general, the use of human‐shaped icons would be too upsetting and that abstract representations were preferable. The site was perceived to be trustworthy though this was in large part because of its affiliation to the UK's National Health Service.

***Scepticism about statistics and predictions***: A common response was to question the accuracy of the predictions. This scepticism was a result of the perception that the tool did not ask for all the possible relevant information (e.g., exercise levels or type of surgery). Participants felt, therefore, that where their individual circumstances were not taken into account by the tool, this limited its ability to provide a useful prediction. A related response was the perception that percentages felt meaningless for an individual. Some participants felt that applying a percentage chance to a categorical event (survival) was hard to understand (e.g., Box 1 comment 4). Another important factor was that other tools appear to provide different estimates. This potentially has the effect of reducing trust in all tools, or leading patients to use those tools that they perceive to provide more optimistic outlooks (e.g., Box 1 comment 3)^1^. Finally, participants felt that the predictions must be based on old data, and therefore that they would not be up to date and include recent developments in treatment (e.g., Box 1 comment 2).

***Information on side effects***: Participants identified that the tool only supplied quantitative information about the benefits of adjuvant therapies, and that they would also want to know about the side effects and their likelihood. Participants felt that in some cases side effects might be severe enough that they would refuse a treatment with only a small benefit. Of particular note was that some patients in the forum indicated surprise at how little potential benefit they were receiving from their treatment regimens (e.g., Box 1 comment 5).

***Interface***: Several aspects of the existing interface were problematic for participants. The primary concern was that small increments were difficult to read on the bar chart (see Figure 1). Many participants in the online survey also stated that the wording 'an extra x women would survive with (treatment)', confusing and would prefer the total number surviving instead.

Table 2 shows the key issues identified from the clinical feedback along with their source. The UKBCG data indicated a majority support for all but two predictors in Predict: Breast Cancer (exercise level and smoking status, see Table S4 for full data). A majority of the respondents supported the introduction of all of the proposed new outputs (Table S5). Survey responses also indicated a wide range of devices were used to access Predict: Breast Cancer, and that it was used in a variety of contexts (full data in Tables S2 and S3). The most common of these was in consultation with patients (80% of respondents), but alone (71%) and MDTs (50%) were also frequent responses. Participants were also able to indicate (via free‐text response) which features of other tools they would like to see included. The features mentioned at least three times were: disease‐free survival (3), outputs to share with patients (3), mobile‐friendly interface (3), cancer recurrence information (9), cancer‐specific mortality (9) and co‐morbidities (20).

**TABLE 2:** Major design requirements derived from background research with clinicians

Issue	Source	Requirements
Time per patient typically around 3 min	MDT observation	Ability to generate results quickly
Importance of not carrying over parameters from previous patient	MDT observation	Ability to reset interface
Site is most useful when predicted increase in survival is small (3%–5%)	MDT observation	Ability to inspect small increments with precision
New predictors requested	Clinician survey (Table S4); Clinician focus group	Implement new predictors or add to future research programme
New outputs requested	Clinician survey (Table S5); Clinician focus group	Implement new outputs or add to future research programme
Keep interface simple	Clinician survey	Maximise speed and ease of use
Variety of different use cases (e.g., teaching, patient consultations and MDTs)	Clinician survey; Clinician focus group	Create outputs and visualisations to support each use
Unusual technical requirements in some hospitals	Clinician focus group	Ensure backward compatibility with browsers no longer supported by manufacturer, and enable offline use
Patients may not have ability to access online tools	Clinician focus group	Print function (with graphics being clear in grey scale)

Clinician survey: 75 respondents at the UK Breast Cancer Group Meeting 2016. Clinician focus group: 18 clinicians at the Cambridge breast unit. See methods for further details.

The UKBCG survey data provided good validation of the clinician focus group feedback. The proposed new outputs all received support, as did the majority of proposed predictors. The free‐text responses also raised similar issues, including technical IT requirements, sharing of outputs with patients and the value of recurrence forecasts.

The following themes identify and expand on commonalities identified in Table 2:

***Simplicity***: Clinicians were very keen that the tool should be quick to use and with minimal constraints on access. This included highlighting technical restrictions in hospitals such as poor WiFi coverage and outdated browsers. Feedback also emphasised that the site should not require users to log in or demand inputs that might not be available. 'Keep it simple' was a sentiment expressed in the focus group and survey comments (there were four unsolicited comments to this effect in the survey), and simplicity was a much appreciated feature of the original interface.

***Additional functionality***: Many clinicians were keen to see new treatments added to the prognostic model (e.g., radiotherapy and bisphosphonates, see also Table S4). Clinicians also highlighted additional outputs that would be useful such as different time frames for the model's predictions. Clinicians also reported that not all patients would have access to computers and internet, meaning the ability to print the outputs was particularly useful.

***Different use cases***: Observation of the MDT and feedback from clinicians highlighted the variety of contexts in which the tool is used. In MDTs the tool was most often used in cases whether it was unclear if a sufficiently large increase in survival would be obtained. Consequently, the ability to inspect small increments in the tool's output was critical. Clinicians also used the tool with patients during consultations to help explain the benefits of treatments. Additional uses identified from the survey included research, teaching and meeting preparation.

### 3.2 Interface development

The new Predict: Breast Cancer website can be visited at the following address: https://breast.predict.nhs.uk/ and the open‐source code is available here: https://github.com/WintonCentre/predict‐v21‐main

Clinician feedback on the wireframe prototype was used to confirm or reject different features that we proposed in response to the background research. In addition, new issues were identified that were implemented in the next iteration where this was possible. Where it was not possible to implement requested features, they were recorded for future implementation. Table S6 in the Supplementary Materials outlines the assessment of the various features in more detail.

A fully functioning online prototype was built that incorporated the features approved, or identified for implementation, in the feedback on the wireframe interfaces. The functioning prototype was then submitted to usability testing. The analysis of usability testing data with patients and clinicians revealed two broad categories of issue. The first was confusion over the subtler functionality of the interface, such as not realising that that the information icons were clickable or failing to notice a button.

The second category concerned how the patients' personal circumstances would map on to the input fields available on the site. For instance, participants wanted to know why the interface does not differentiate between the types of surgery a patient can have (e.g., lumpectomy or mastectomy), or what to do when they lacked sufficient information to fill in all the input fields.

**FIGURE 2:**
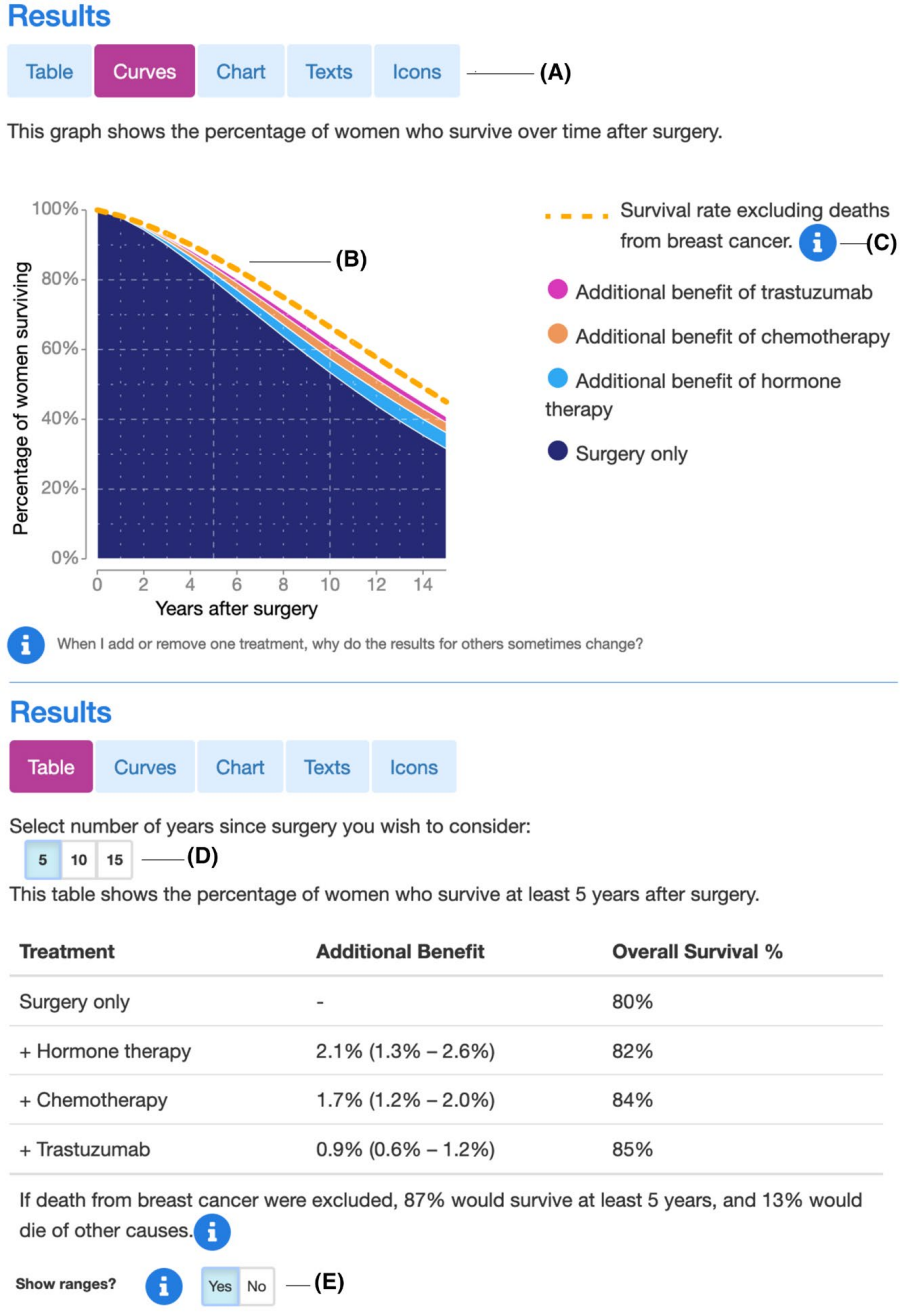
Display options for the new Predict: Breast Cancer website showing a survival curve in the top panel and a tabular format in the bottom panel. Users can choose from these formats and three others. (A) choice of display, (B) survival rate excluding breast cancer, (C) information icons, (D) choice of timeframe and (E) optional prediction ranges. Figure 3 shows the other three display options

**FIGURE 3:**
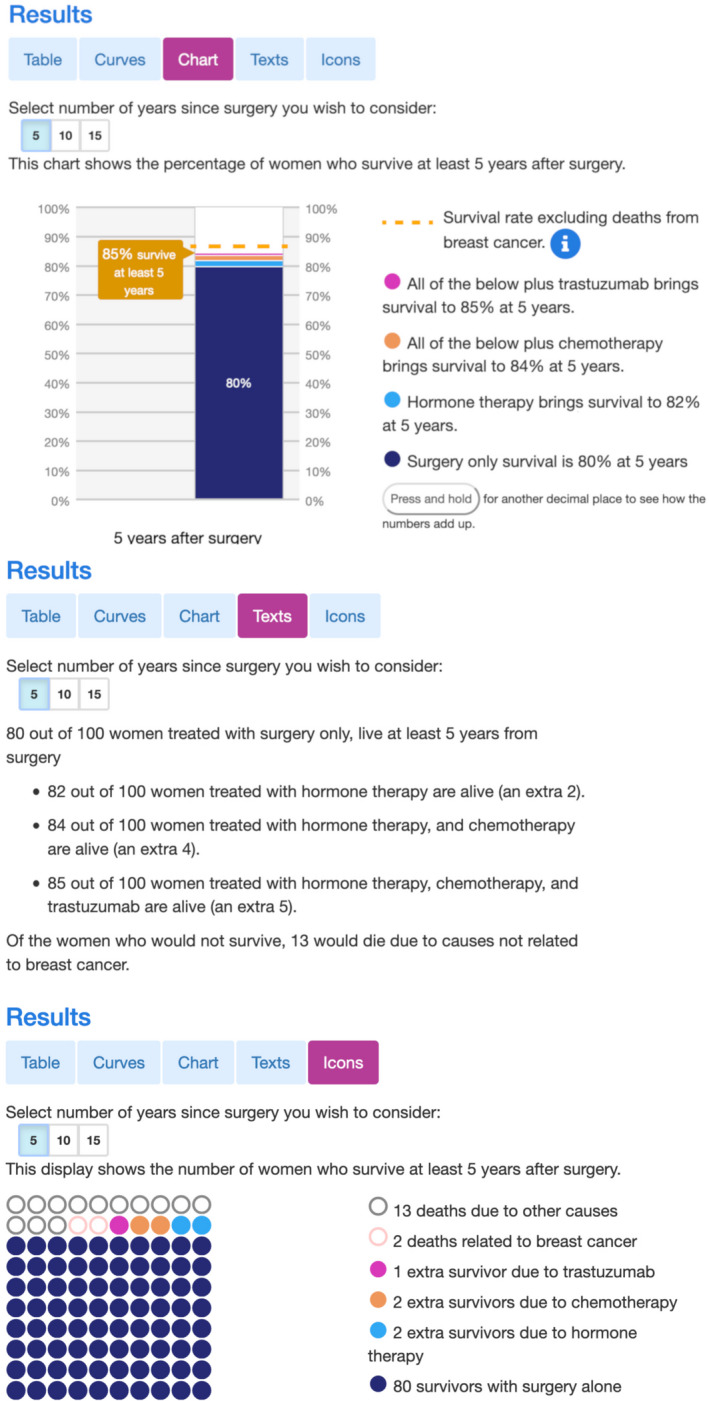
Three different display options for the new Predict: Breast Cancer website. The top panel shows a stacked bar chart, the middle panel a text representation of the same results and the bottom panel shows an icon array. Users can choose of which these displays they want to use to see the results. The two other display options are shown in Figure 2

As issues were identified they were addressed through changes to the interface. These were predominantly adding more explanatory information text, and alterations to the layout and prominence of interactive elements. Figures 2 and 3 shows the display incorporating these changes. See Supplementary Materials, for example, data on the issues raised during usability testing and their solutions (Table S7).

Our approach to conflicting feedback was to try and accommodate both points of view through compromise. For example, we received mixed feedback about the use of uncertainty ranges. These were included but turned off by default so as to not overwhelm users who might find them difficult to interpret. Although led by feedback from users, final decisions about the interface were made by the authors.

With respect to the default settings of the interface, based on participant feedback, we erred on the side of the simplest presentation possible. For instance, with the decimal places on predictions, by default the interface presents a whole number, but interested users can press a button to see more precise values. We also added flexibility to the default settings such that users can open a settings menu and choose which of the presentation formats they want the display to default to. This menu also allows Bisphosphonates to be removed from the treatment options, as it is not available in all geographic regions.

#### 3.2.1 New features

The finished interface includes many new features derived from the background research and feedback during iterative development process. These include: The option to choose a preferred method for displaying the results (Figure 2A), the inclusion of non‐breast cancer mortality (Figure 2B), clickable information icons which display explanations of input fields and outputs (Figure 2C), the ability to specify the time range over which to display the predictions (Figure 2D) and the optional provision of precision estimates around the predictions (Figure 2E).

The research and design process also revealed additional predictors and treatments that patients and clinicians would like to see as part of the model. Data were available for some of these, consequently bisphosphonates and extended hormone therapy have been added as treatments, whilst menopausal status has been added as a predictor. The Predict: Breast Cancer site now also has updated FAQs and technical information pages for non‐experts. The site has been translated into Spanish, French, Portuguese and Chinese, and a graphic designer was employed to create a coherent professional appearance for the site.

#### 3.2.2 Future features

The design process allowed us to identify additional predictors and outputs that clinicians and their patients would find useful. These data provide a valuable insight into how users would like the site to evolve, and they form the basis for a plan of future research and development. Crucially, these development plans are user‐driven and reflect how patients and clinicians would like the algorithm and interface to work in order to be maximally useful.

One requested function was the ability to use the tool for estimating the impact on survival of *stopping* adjuvant treatments. Patients may request this information because the side effects of treatments are often unpleasant enough that they want to re‐assess whether the treatments are worth continuing with.

A common query amongst patients was how to gauge the likelihood and severity of side effects. Whilst qualitative information on side effects is available, there is very little quantitative information. Work is now underway to provide these data.

A related issue is the increase in mortality that some treatments cause. This can, in some patients, outweigh any benefit, meaning the treatment would result in a drop in predicted survival rates. This vitally important component is not currently included in Predict: Breast Cancer, and research is underway to determine how best to display such a 'negative benefit' unambiguously in each of the graphical formats used in Predict: Breast Cancer.

Two frequently requested features were co‐morbidities as a patient input, and radiotherapy as a treatment option. The data are not currently available to model the effects of co‐morbidities. However, radiotherapy as a treatment option is being modelled and will be added to a future release of the site.

Finally, both clinicians and patients expressed the desire to see the chances of breast cancer recurrence as well as survival. This can be modelled, and a programme of work is underway on how best to visualise this.

## 4 DISCUSSION

Our background research revealed a number of themes which we addressed in the design of a new interface for Predict: Breast Cancer. For our non‐clinician participants these included: Fear of a poor prognosis, appearance and trustworthiness, scepticism about statistics, information on side effects and problems with the interface. The background research with clinicians revealed a desire for simplicity, additional functionality and the need to cater for several different use cases. These themes reflect the differing needs of patients and clinicians. For patients, they reflect the fact they are dealing with new information that is difficult is to absorb, both in terms of its emotional impact and its complexity. The clinician group was more expert and, therefore, interested in increased functionality and flexibility, as long as this this did not come at the cost of speed and simplicity. In this particular case, simplicity centred on not obstructing use of the tool by requiring logins, mandatory input fields or recent browser technology. As with simplicity, catering for different contexts of use is a fundamental principle of UCD^12^, ^20^ but can be easily overlooked.

The broad requirements identified in the background research resulted in initial prototypes that were iteratively refined with feedback from clinicians and patients. Usability testing then made sure that our proposed solutions were effective and usable. The end result was the new interface to Predict: Breast Cancer which is now live.

During this research, we identified some key recommendations for developing public or clinician‐facing prognostic algorithms (see Box 2). The first of these is to make interactions flexible (R1). This means not prescribing to users how they should use the tool, instead building in enough flexibility so that different users can interact in different ways. For the new Predict: Breast Cancer interface we tested features that would allow a side by side comparison of treatments. Ultimately, however, users preferred to change the parameters and observe how the displays changed. Rather than prescribe how users should compare treatments we made the displays responsive so that they updated immediately upon a change to the predictors or treatments.

BOX 2 **Recommendations for communicating with prognostic models**

The following recommendations have been derived from our experience of developing the new Predict: Breast Cancer interface. Each is addressed in more detail in the discussion.

R1. *Make interactions flexible*: It is important for an interface to allow the user to interact with the model's predictions in a way that suits their needs. Rather than restrict or prescribe how the user should behave, try to build in flexibility. This maximises the chances of catering to different use cases.

R2. *Put statistics in context*: It is possible to communicate numbers perfectly accurately but leave the recipient none the wiser. The provision of contextual information can be critical in turning a number into information. Many users, especially patients, need a frame of reference to meaningfully evaluate a prediction.

R3. *Identify and address gaps in knowledge*: It is important to address potential misunderstandings explicitly. For patients and clinicians, supporting information helps ensure the data is input correctly and therefore increases confidence in the results.

R4. *Provide the downsides*: People want to understand the negatives as well as the positives. When using models to provide the benefits of treatments, consider that many patients will want to be able to trade‐off that information with the likelihood and severity of side‐effects.

R5. *Generate a programme of research*: During background research and user testing of a prognostic model, many new predictors and potential outputs will be suggested. Where these cannot be implemented (due to lack of data, for example) they should nonetheless be recorded as they provide a valuable source of user‐driven future research projects.

R6. *Engage with prospective users*: The most important recommendation is to obtain as much feedback from the target audience as possible. Each prognostic model will be unique, and user centred design reveals vital requirements that are often unforeseeable without consulting users.

A further example of this approach was our inclusion of multiple ways of displaying the results, thereby allowing the user to select whichever format best suited their purposes. Whereas tables facilitate MDT inspection of small increments, survival curves allow patients in consultations to see the how the benefits of treatments change over time. We also found that users liked being able to compare different formats as a means of checking their comprehension. The use of multiple presentation formats has previously been identified as beneficial for patient understanding in a review of the literature on prognosis communication.^21^

Our second recommendation, to *put statistics in context* (R2) is implemented in our inclusion of the survival rates for women who do not die of breast cancer. This information provides critical context by putting an upper bound on the possible benefit of a treatment. A survival probability of 60% may seem less frightening in the context of a survival probability of 70% for women who do not die of breast cancer. And the maximum possible increase of 10% provides context in evaluating the efficacy of a treatment that improves survival by 5%. Other information, such as uncertainty estimates, allow the tool to convey not just the point estimate for survival (e.g., 60%) but also a range indicating the likely variability around the estimate, (e.g., 57%–63%), especially important to consider when the potential benefits are small.

The provision of context has wide support in the literature on risk communication and is a key recommendation in the recently updated International Patient Decision Aid Standard (IPDAS)^22^ as well as in a number of reviews on risk communication.^13^, ^23^ A key concept here is that of evaluability^24^ which relates to whether a person can assess the information as good or bad, and therefore whether it is useful to decision‐making. Changing the evaluability of information has been shown to lead to preference reversals, including medical contexts.^25^ Unfamiliar medical information may be especially difficult to evaluate for patients, and hence the importance of contextualising such information. The use of uncertainty ranges on estimates was regarded as beneficial for clinicians, and whilst some patients were interested, it should be noted that they can be difficult to interpret for non‐specialists.^22^

The recommendation to *address gaps in knowledge (R3)* is about ensuring the predictors are entered correctly and the users (both clinicians and patients) have confidence in the resulting predictions. Our background research and usability testing identified instances of both clinicians and patients being unsure of what was being asked of them. This is an issue that has previously been identified in the context of prognostic models, where the applicability of a tool can be limited through ambiguity in what is being asked for in a predictor.^26^ In addition to ensuring predictors are entered accurately, previous research has also identified that clarity about how the algorithm functions is important in increasing users' confidence in the results.^27^ In our interface this was in part achieved by adding FAQ and technical information sections to the website that used lay language where possible. The interface also includes information buttons that when selected launch a pop‐up window providing a detailed explanation of the predictor being requested or the output being presented.

In our research it quickly became apparent that users would want to evaluate any increase in survival against the potential for side effects. Our recommendation to *provide the downsides (R4)* is not something we could immediately implement in Predict: Breast Cancer as quantitative data on the probability and severity of side effects is not readily available. Nonetheless developers of other models should investigate whether such information is available in their domains. This theme relates to Recommendation 2 in that it is of course difficult to evaluate a treatment if only the benefits are presented. The desire amongst patients for information on negative effects is also supported in the literature on prognosis communication.^21^ It may be that patients would prefer not to have treatment with a small average reduction in mortality if it means substantially lowering quality of life. For those who take treatment, awareness of the likelihood and severity of potential side effects (and ways to ameliorate them) could help increase adherence to the treatment and reduce worry about symptoms that arise.

The research to provide quantitative data on the side effects of treatments shown in Predict: Breast Cancer is underway, and provides the basis for our recommendation to *Generate a programme of research (R5)*. Many more suggestions for predictors and features in Predict: Breast Cancer were made than we could implement. However, these suggestions remain a valuable record of what users would like to have access to. For popular requests that cannot be met, perhaps due to a lack of data, researchers have a ready‐made project with excellent chances of impact.

The last, and most important, recommendation is to *Engage with prospective users (R6)*. This is fundamental to maximising the impact that a model can have. However, statistically sound and accurate a model is, if it cannot be used by its intended audience, or lacks some critical feature, it will not achieve the impact it otherwise could.

Engaging with users at all stages of the design will identify issues that cannot be foreseen. For Predict: Breast Cancer we had to conduct the background research in order to understand the different contexts of use and critical technical requirements for the site to continue working on hospital IT systems. We also did not foresee that, although recommended in the risk communication literature,^13^, ^28^, ^29^ icon arrays would be deemed to be distressingly morbid when human‐shaped. More generally, the need for research on side effects, the extent of the supporting information required by users and the new predictors and outputs, were all based on feedback from prospective users. These features and outputs would likely have been missed or underestimated without their input. An equally important aspect of UCD is determining what does not work––without it, we would certainly have included features that were either not used, or worse, misunderstood.

Consulting with users is increasingly recognised as an important step and is an overarching principle in the IPDAS recommendations for communicating probabilities to patients.^22^ The full UCD process extends this idea to the whole interface. UCD is comprised of many more techniques and methods than we employed in the development of the interface for Predict: Breast Cancer. Which methods are used will depend on the target audience, resources available and the expertise of the researchers involved. However, the fundamental process of UCD is straightforward: Engage in background research, then iteratively test and refine designs with the target audience. Even if resources are very limited, there is still value in engaging in the process to whatever extent is possible.

The new Predict: Breast Cancer website is live and delivering thousands of sessions worldwide per week. Its development was based on a combination of broad background research and user‐testing with the target audiences. By involving users in the design process, we believe it has enhanced the potential for shared decision‐making and informed consent. Furthermore, the interface has now been successfully applied to a similar prognostic algorithm for prostate cancer (https://prostate.predict.nhs.uk) where its effects on decision‐making and patient satisfaction are being evaluated in clinic.^30^, ^31^

### 4.1 Study limitations

To ensure the effectiveness of design decisions there is no substitute for feedback from prospective users in the development process. The extent to which this is possible will vary from case to case. Ideally, in our case, this would have involved working with patients who were in the process of making decisions about adjuvant treatments in all stages of the interface development. We were only able to recruit a limited number of patients and we deemed the usability testing the most valuable time to involve them. Our background research would have been improved by a greater involvement of patients. Although our participants recruited from the general public gave us useful insights into how Predict: Breast Cancer is perceived by a first time user, they were not having to process the information with the distress caused by a recent cancer diagnosis.

### 4.2 Clinical implications

Epidemiological models can provide vital information for clinicians and patients deciding on which treatments to take. This in turn can lead to increased survival rates and all‐round improved decision‐making. UCD can remove the barriers to these benefits and, by engaging with patients, set an agenda for future development that really addresses their needs. In the case of Predict: Breast Cancer this has led to the first quantitative review of side effects for many adjuvant therapies and additional modelling to address the requested additional treatments and outputs.

### 4.3 Conclusions

Prognostic algorithms have great potential for improving shared decision‐making and informed consent. If made available in clinic, they can ensure that patients and healthcare professionals receive accurate and relevant information about the personalised benefits and harms of different treatment options. In order to make such complex and emotionally difficult information available in a clear, unambiguous and sensitive manner, UCD is critical. Algorithms need to be statistically validated, but must also be easy to use, trustworthy and produce outputs that are clear and useful to their users.

## CONFLICT OF INTEREST

No conflict of interest to report.

## ETHICS STATEMENT

This study was approved by the University of Cambridge Psychology Research Ethics Committee (PRE.2016.103). Participants gave informed consent before taking part.

## Supporting information

Supplementary Material

## Data Availability

The data that support the findings of this study are available from the corresponding author upon reasonable request.

## ENDNOTE

^1^
Major discrepancies were due to some tools excluding mortality not related to breast cancer.
