# Supplementary material for: Redevelopment of the Predict: Breast Cancer website and recommendations for developing interfaces to support decision‐making
Source: Cancer Med. 2021 Jun 21;10(15):5141–53. doi: 10.1002/cam4.4072 (PMC8335820; doi:10.1002/cam4.4072)
Supplement: Supplementary file 1 — Supplementary Material [file CAM4-10-5141-s001.docx]

# Supplementary Materials

| **Table S1: Ratings from the public survey of the original Predict:Breast Cancer site** | | | | | |
| --- | --- | --- | --- | --- | --- |
| **Topic** |  | | | | |
|  | **Completely Disagree** | **Somewhat Disagree** | **Neither** | **Somewhat Agree** | **Completely Agree** |
| I thought the appearance (pictures, colours, fonts etc) of the website was appropriate | 8% | 14% | 14% | 36% | 28% |
| I would trust that the results are accurate | 0% | 4% | 14% | 58% | 24% |
| I would find the results graph easy to understand | 2% | 18% | 10% | 36% | 34% |
| would find the text* results easy to understand  *(e.g., x out 100 women are alive at 10 years) | 0% | 16% | 4% | 36% | 44% |

Participants also gave the site an overall rating: Very poor (0%), Poor(10%), Neutral (22%), Good (40%), Very good (28%)

Tables S2 to S5 show data from surveys distributed to attendees at the UK Breast Cancer Group meeting in 2016. Seventy-five surveys were returned.

| **Table S2. Devices used to access the Predict:Breast Cancer website (72 responses)** | |
| --- | --- |
| **Device** | **Percentage of respondents that use this device** |
| Desktop | 80.6 |
| Laptop | 37.5 |
| Mobile | 31.9 |
| Tablet | 18.1 |

| **Table S3. Contexts in which Predict:Breast Cancer is used (72 responses)** | |
| --- | --- |
| **Context** | **Percentage of respondents that use Predict in this context** |
| With patients | 80.6 |
| Alone | 70.8 |
| MDTs | 50.0 |
| Teaching | 40.3 |
| Research | 5.6 |

| **Table S4. Assessment of potential predictors (32 responses)** | |
| --- | --- |
| **Predictor** | **Percentage of respondents that do not require** |
| Exercise Level | 78.1 |
| Smoking Status | 53.1 |
| Body Mass Index | 40.6 |
| Ki-67 | 31.3 |
| Oophorectomy | 21.9 |
| Neoadjuvant outcomes | 9.4 |
| PR Status | 6.3 |
| Oncotype/Mammoprint | 6.3 |
| Bisphosphonates | 3.1 |
| Vascular Invasion | 3.1 |
| ER Status | 0 |
| HER2 | 0 |
| Radiotherapy | 0 |
| Lymph node involvement | 0 |

| **Table S5: Responses by clinicians surveyed at the UK Breast Cancer Group meeting 2016: Ratings for or against the inclusion of new outputs (22 responses)** | | | | | |
| --- | --- | --- | --- | --- | --- |
| **Potential outputs** | **Responses** | | | | |
|  | **-2 (No)** | **-1** | **0** | **+1** | **+2 (Yes)** |
| Option to display full survival curve beyond 10 years | 0% | 4.8% | 0% | 14.3% | 81% |
| Breakdown of mortality/disease caused by cancer treatments | 0% | 0% | 0% | 10% | 90% |
| Adding error bars to illustrate degree of uncertainty around the results | 9.1% | 4.5% | 0% | 9.1% | 77.3% |
| Option to display table with numbers rather than graphical display | 22.2% | 0% | 5.6% | 11.1% | 61.1% |
| Options to display disease-specific mortality (not all cause mortality) | 4.5% | 0% | 0% | 9.1% | 86.4% |
| Links for patients to information about short term adverse effects | 5% | 0% | 5% | 15% | 75% |

| **Table S6: Key feedback from clinicians on early prototype** | |
| --- | --- |
| **Feature / Problem** | **Assessment / *Action*** |
| Feature: User can choose preferred visualisation of output (bar chart, survival curve, icon array, table & text) | Useful for different contexts (MDT, consultation), but also likely to enhance overall understanding via comparison of displays. *Carried forward* |
| Feature: Visualisations update immediately upon change to inputs or treatments | Useful for assessing and contrasting contributions of individual treatments. *Carried forward* |
| Feature: Ability to select different treatment regimens and compare outputs side by side | Deemed unnecessary, as function could be fulfilled by having an interface that updates immediately (see row above). *Removed* |
| Feature: Display margins of error on survival estimates | Useful, but cautious that patients will find confusing, or interpret results as less reliable. Maybe used by clinicians to convince patients to accept or reject treatment. *Carried forward* |
| Feature: User can choose time frame for estimated survival | Especially useful for consultations with patients. Previous presence of two bar graphs showing 5 and 10-year predictions was confusing and not always appropriate depending on patient’s prognosis. *Carried forward* |
| Feature: Non breast cancer mortality | Important contextual information. Especially for older patients, this helps put the impact of the cancer and treatments into perspective. *Carried forward* |
| Problem: Clinicians concerned that mandatory inputs might prevent them using the tool if they do not have the necessary information. | Requirement that additional inputs have an ‘unknown’ option unless they are critical to the algorithm’s prediction. *Implemented in next iteration.* |
| Problem: Patients do not always have technical skills or resources to use online tools | Requirement that print-outs are available showing all visualisations. Colours need to print clearly in grey scale. *Implemented in next iteration* |
| Problem: Clinicians (and their patients) want reassurance that predictions are up to date and accurate. | Requirement to provide information in lay language on how the algorithm is validated and updated. *Addressed with FAQ and ‘about’ text in next iteration.* |
| Problem: The order in which the treatment benefits are displayed affects the size of the predicted benefit. This can run counter to the benefits clinicians expect from published trials and their experience | Requirement to explain why treatment benefits may not match clinicians’ expectations. *Implemented in next iteration* |
| Problem: Patients often want to understand the impact on their prognosis if they stop adjuvant treatments | Requirement for algorithm to model stopping treatment and display the appropriate output. *Added to future work.* |
| Problem: Patients want to understand the probability of recurrence separately from mortality | Requirement for algorithm to model recurrence and display the appropriate output. *Added to future work*. |
| Problem: Clinicians often need to use PREDICT in hospitals where Wi-Fi and phone signal is intermittent | Requirement to develop version of the site that works like an app on mobile devices without the need for an internet connection. *Implemented for final version* |

Table S7 provides examples of issues identified during usability testing with patients and clinicians. Participants used a functioning prototype while their interactions where recorded.

| **Table S7. Examples of issues identified during usability testing and their solutions** | |
| --- | --- |
| **Issue** | **How addressed** |
| Participants don’t always realise there are different tabs with different visualisations | Colour scheme changed to make the tabs more obvious |
| Patients want to read page entitled “information for professionals” | Renamed “technical information” so as not to make patients feel excluded. Reworded to remove jargon |
| People have questions about specific features of tumours not available as inputs | FAQs added to make it clear which types of tumour were included in PREDICT. (invasive, not ductal carcinoma in situ, nor lobular carcinoma in situ) |
| Patients do not recognize the generic drug name trastuzumab | Information box created to give more details on each treatment option, including labelling trastuzumab as ‘Herceptin’. |
| ‘Bubbles’ highlighting incremental benefit confusing & obscuring graphic | Only one bubble kept – other information put into legend or on chart |
| Participants don’t find bar charts side by side with different timeframes helpful | Dual display changed and replaced with single chart and options for how many years post-surgery to display |
| Arrays too small | Icon size increased. |
| Some wonder whether the results in the table are cumulative. | Cumulative total added as a column |
| It is not always clear that people can select different timeframes | Button changed to be more obvious, placed in the middle of the introductory text. |
| More guidance required about what to do with parameters that are not a neat fit to the options given. | Guidance given in information buttons about how to deal with more complicated inputs. |
| Grey line (non breast cancer mortality) not always noticed in graphs, not always noticed in table either. | Line made orange and format of table changed to make it more obvious. |
| Confusion over what non breast cancer mortality line represents | New wording attempted: “if nobody died from breast cancer” |
| Many people are keen to see uncertainty ranges, but don’t want to complicate the interface. | Not displayed by default but added as an option |
